# The Glycan Structure of *T. cruzi* mucins Depends on the Host. Insights on the Chameleonic Galactose

**DOI:** 10.3390/molecules25173913

**Published:** 2020-08-27

**Authors:** María Eugenia Giorgi, Rosa M. de Lederkremer

**Affiliations:** Departamento de Química Orgánica, Facultad de Ciencias Exactas y Naturales, Centro de Investigaciones en Hidratos de Carbono (CIHIDECAR), Consejo Nacional de Investigaciones Científicas y Técnicas, Universidad de Buenos Aires, Buenos Aires 1428, Argentina; megiorgi@qo.fcen.uba.ar

**Keywords:** *Trypanosoma cruzi*, mucins, β-galactofuranose, α-galactopyranose

## Abstract

*Trypanosoma cruzi*, the protozoa that causes Chagas disease in humans, is transmitted by insects from the Reduviidae family. The parasite has developed the ability to change the structure of the surface molecules, depending on the host. Among them, the mucins are the most abundant glycoproteins. Structural studies have focused on the epimastigotes and metacyclic trypomastigotes that colonize the insect, and on the mammal trypomastigotes. The carbohydrate in the mucins fulfills crucial functions, the most important of which being the accepting of sialic acid from the host, a process catalyzed by the unique parasite trans-sialidase. The sialylation of the parasite influences the immune response on infection. The *O*-linked sugars have characteristics that differentiate them from human mucins. One of them is the linkage to the polypeptide chain by the hexosamine, GlcNAc, instead of GalNAc. The main monosaccharide in the mucins oligosaccharides is galactose, and this may be present in three configurations. Whereas β-d-galactopyranose (β-Gal*p*) was found in the insect and the human stages of *Trypanosoma cruzi*, β-d-galactofuranose (β-Gal*f*) is present only in the mucins of some strains of epimastigotes and α-d-galactopyranose (α-Gal*p*) characterizes the mucins of the bloodstream trypomastigotes. The two last configurations confer high antigenic properties. In this review we discuss the different structures found and we pose the questions that still need investigation on the exchange of the configurations of galactose.

## 1. Introduction

*Trypanosoma cruzi*, the agent of Chagas disease [1], is an intriguing parasite, not only because of the morphological and biological changes during its life cycle but also for the drastic modifications of the sugars at the surface of the parasite. The multiple strains of *T. cruzi* have been grouped into seven discrete typing units (DTU) (TcI to TcVI and Tcbat) based on their phenotypic and genetic properties [2,3].

**Figure 1:**
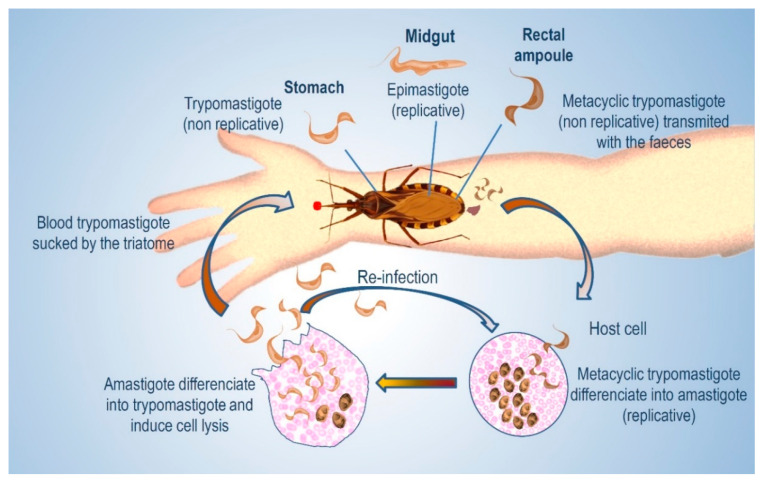
Life cycle of *T. cruzi*.

On the other hand, four main stages can be recognized in *T. cruzi*, depending on the host being either the triatomine insect or a mammal [4,5,6]. In each host a replicative and an infective form have been described. The replicative forms are the epimastigotes in the insect and amastigotes in the mammal, whereas trypomastigotes (metacyclic in the insect) are the infective forms. (Figure 1)

The transmission to mammals occurs when the triatomines, while feeding, deposit feces on the skin, and parasites penetrate through the small wounds caused by scratching. Several factors influence the success of infection [7]. Oral transmission of *T. cruzi* followed by mucosa infection has also been characterized as highly lethal [8,9]. The mechanisms of exocytosis and endocytosis that take place in the host and favor *T. cruzi* invasion were studied [10]. Before cell invasion, there is an interaction of the parasite with the extracellular matrix that results in metabolic modifications. Although cell-derived trypomastigotes were used in the study, it is conceivable that changes also occur in insect-derived metacyclic trypomastigotes [11]. Once in the cell, trypomastigotes accumulate in a parasitophorus vacuole (PV) formed by fusion with the host lysosome, an essential step in order to evade the immune response of the host [12,13]. Several biological processes take place in the PV, and one of them is due to the action of the *T. cruzi* trans-sialidase (TcTS), referred later in this review, which mediates parasite escape from PVs to cytosol [14,15]. In the PV, the parasites start to transform into amastigotes, and differentiation is completed in the cytosol. After several cycles of binary division, they differentiate again into trypomastigotes, which upon membrane lysis are released to circulation. These blood trypomastigotes may infect other cells or be ingested by a triatomine. In the insect, the trypomastigotes must differentiate into epimastigotes to close the cycle. This process starts in the stomach; down in the rich medium of the midgut they multiply, and upon reaching the hindgut they transform into the infective metacyclic trypomastigotes, which detach from the cuticle and are excreted.

Regarding the polypeptide chains in the mucins, multiple genes encoding mucins have been described and grouped into families depending on the host, which may be an insect or a mammal. Studies from expert groups on the genes and proteins of *T. cruzi* mucins have been published [16,17,18,19,20,21]. A synthetic peptide from a mucin associated surface protein (MASP) was proposed as a candidate for a vaccine against Chagas disease [22].

The parasites, in the developmental stages identified in *T. cruzi*, express characteristic molecules that are crucial in the infection. However, the metabolic steps involved in their fate during transformations are poorly understood. The recognition of surface glycans by host cells is well documented [23,24,25]. In this review we will focus on the structure and role of the glycans at the surface of the parasite in the different developmental stages, mainly epimastigotes and trypomastigotes, because studies on glycans of amastigotes are scarce. A conclusion, with an addressing of the questions that arise from the current knowledge, is included.

## 2. Cell-Surface Glycans in *T. cruzi*

**Figure 2:**
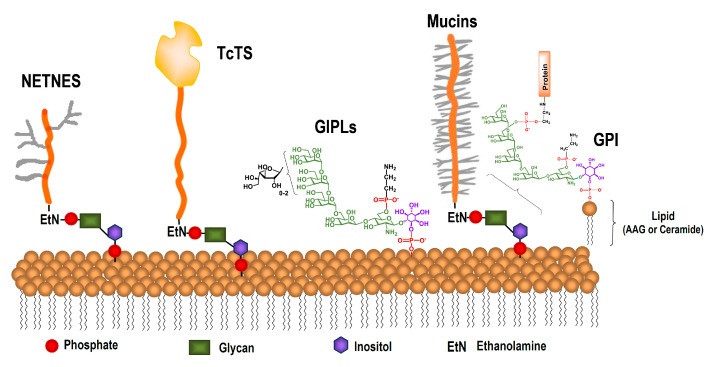
Representative glycoconjugates in the surface of *T. cruzi* epimastigotes.

The most abundant molecules in the glycocalix that covers the parasite are glycoinositolphospholipids (GIPLs) [26,27] and glycosylphosphatidylinositol (GPI)-anchored mucins [28,29] (Figure 2). The GPI favors the dense packing of the mucins. Epimastigotes and trypomastigotes have approximately the same number of mucin molecules per cell [30], whereas a significantly higher number of GIPL molecules was detected in epimastigotes [31]. Other less abundant but unique glycoproteins have been described in *T. cruzi*, among them a trans-sialidase, [32,33,34] and in epimastigotes, a complex GPI-anchored glycopeptide called NETNES [35].

## 3. The Glycan in Mucins of *T. cruzi*

**Figure 3:**
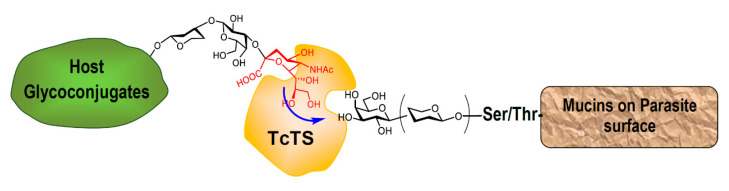
Trans-sialidase from *T. cruzi* transfers sialic acid from host sialoglycoconjugates to β-Gal*p*-containing glycoproteins expressed on the parasite’s surface.

The carbohydrate in the mucins amounts to about 60% of the total mass [30]. The structure of the *O*-linked chains in the mucins defines their role in antigenicity and pathogenesis. They perform a crucial function as acceptors of sialic acid from host glycoconjugates in a reaction catalyzed by the parasite’s unique trans-sialidase (TcTS), an enzyme extensively studied. The negatively charged glycans protect the parasite from the action of proteases and other enzymes [32,34,36,37]. This is the only way for *T. cruzi* to acquire the sialic acid needed for infection [15,38]. The reaction depends on the structure of the *O*-linked sugars in the mucins; more precisely, on the presence of β-Gal*p* terminal units (Figure 3). Sialylation affects the sialoglyco-profile of both the parasite and the mammal host, thus modulating immunological events. The natural change of one aminoacid in the peptidic chain leads to the inactivation of the enzyme; however, this inactive trans-sialidase (iTcTS) shows lectin properties in relation to Neu5Ac (α2-3-Gal) glycotopes influencing adhesion and invasion of the host cell [15,39].

**Figure 4:**
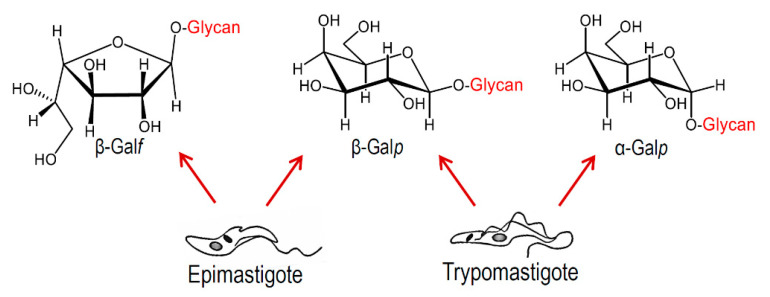
Configurations of Galactose (Gal) found in *T. cruzi* mucins.

As will be discussed below, the *O*-linked carbohydrate chains display great variability, and galactose being the main monosaccharide constituent, it may be present as β-galactofuranoside or as galactopyranoside in α- and/or β- anomeric configurations, depending on the strain and the stage of the parasite studied (Figure 4). The microheterogeneity and highly diverse structures of the *O*-linked glycans have been described. The higher amount of β-Gal*p* terminal units, available for sialylation, may determine the virulence of the parasite. On the other hand, β-Gal*f* and α-Gal*p* are highly antigenic, and thus could provoke an immune response by the host.

The glycan structures of human and *T. cruzi* mucins show striking differences [40]. First, the sugar chains are linked to the protein by α-GlcNAc instead of GalNAc, like in vertebrate mucins [41,42]. Moreover, apparently GalNAc was not found in *T. cruzi* glycoconjugates. Accordingly, the UDP-Glc4′-epimerase is unable to convert UDP-GlcNAc to UDPGalNAc, in contrast to the human epimerase [43]. The gene encoding the transferase which incorporates the α-GlcNAc from the nucleotide was identified and called TcOGNT-2 [44]. This gene is not equally expressed in all stages of the life cycle of *T. cruzi*. On the differentiation of trypomastigotes to amastigotes inside the mammal cell, the expression levels of TcOGNT-2 decrease and increase again when, after replication, the amastigotes differentiate again into trypomastigotes [45]. Several studies have been published on the sugar structures of mucins, particularly those of epimastigote strains [19,46,47,48,49,50,51]. Unusual for surface glycoproteins, in some strains, a significant amount of non-substituted *O*-linked GlcNAc, which amounted to about 20% of the glycosylation sites, was found [47,52]. In all of them, the next sugar added is galactose, and the disaccharide may be further elongated with galactoses to afford lineal or branched structures [28]. The nucleotide for the incorporation of galactose must be formed from UDPGlc*p* by the action of UDPGlc*p*-4 epimerase, since a galactose transporter could not be identified [53]. The suppression of the epimerase activity caused important changes in the morphology and membrane cell structure of the parasite [54].

The configuration of galactose in epimastigotes varies among strains and from the cell-derived trypomastigotes. Galactofuranose (in the β-configuration) has only been found in the epimastigote mucins of strains belonging to DTU I or hybrid strains, whereas α-Gal*p* is only present in trypomastigote mucins. The other anomer, β-Gal*p*, was identified in all strains of epimastigotes and trypomastigotes, and is recognized by the antibody 2B10 [55]. Apparently, there are no reports on the structure of glycans from amastigote mucins in agreement with the fact that the enzyme that incorporates GlcNAc, the first sugar of the *O*-linked chain, decays upon the differentiation of trypomastigotes into amastigotes [45]. A stage-specific GPI-anchored glycoprotein called Ssp4 has been characterized in extracellular amastigotes. These may be found in the extracellular milieu due to the early lysis of infected cells [56,57], or to cytolysis at sites of infection during the chronic stage of Chagas disease [58]. The extracellular amastigotes share morphological, immunological and infective properties with the intracellular counterparts. β-Galactopyranosides were identified as determinants in Ssp4 of host–cell interactions [59], but the linkage to the protein and the structure of the oligosaccharides were not defined.

## 4. Galactofuranose in Glycoconjugates of *T. cruzi* Epimastigotes

d-galactose in the pyranose configuration is a common constituent of the oligosaccharides, glycoproteins and glycolipids of mammals, whereas Gal*f* is found in bacteria, protozoa and fungi, some of which are pathogenic for humans that lack Gal*f* [60]. In *T. cruzi*, Gal*f* was first detected in GIPLs [61], and there are several reports on its presence in the Leishmania species [62,63]. The selective presence of Gal*f* in mucins of some strains of *T. cruzi* insect forms [28] is particularly interesting; it was not described in *T*. *brucei*. β-Galactofuranosyl-containing conjugates are also constituents of other important human pathogens, like *Mycobacterium tuberculosis* [64] and *Aspergillus fumigatus* [65,66]. Being absent in mammals, the enzymes involved in the biosynthesis of galactofuranosides are good targets for chemotherapy [67]. As with other monosaccharides, Gal*f* is incorporated from the nucleotide UDPGal*f*, which in turn is produced from UDPGal*p* by the action of a mutase (UGM) that was first described in *E. coli* [68,69] and has been extensively studied [70,71]. Candidate genes (GLF) were identified in eukaryotes, among them *T. cruzi*, by combinatorial bioinformatics screening. When GLF were expressed in *E. coli*, the proteins showed UGM activity [72]. The crystal structure of *T. cruzi* UGM showed differences with the bacterial UGMs [73]. The galactofuranosyl transferases (GALFT) working for the introduction of Gal*f* from the nucleotide were less studied. Although genes encoding GALFT have been detected in the genoma of *T. cruzi* [74], the cloning of proteins with enzymatic activity was not reported. Comparative analyses of the amino acid sequence of a GALFT from *T. rangeli* (TrGALFT), a non-pathogenic trypanosome, revealed identities between 73% and 55% with *T. cruzi* orthologs, however antibodies raised against TrGALFT did not recognized proteins in a *T. cruzi* extract [75].

**Figure 5:**
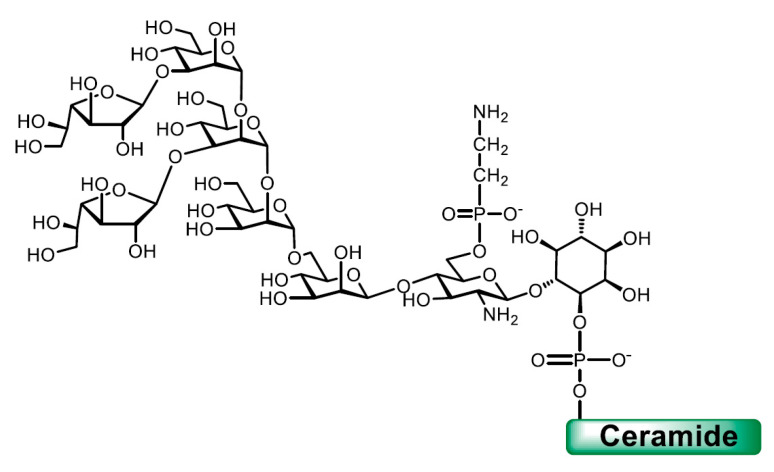
Major structure found in glycoinositolphospholipids (GIPLs) of the Y strain.

In *T. cruzi*, the GIPLs, originally named lipopeptidophophoglycan (LPPG) [76], are the most abundant glycoconjugates in epimastigotes and metacyclic trypomastigotes (10^7^ molecules/cell) [35]. The full structures were determined for GIPLs of different strains [26,27,77]. A microheterogeneity in the glycan structure was described. The major structure (65%), found in GIPLs of the Y strain, with two β-Gal*f* units is shown in Figure 5 [27]. Different from the mucins, as discussed below, Gal*f* was identified in the GIPLs of epimastigote strains belonging to DTU I and DTU II. An exo β-galactofuranosidase able to remove Gal from the GIPLs was reported [78].

In *T. cruzi,* GIPLs, commonly called GPI anchors, are also found attaching proteins like the mucins to the cell membrane [79,80]. However, no Gal*f* was detected in the GPI anchor of mucins from the Y strain [81] or the G strain [47]. In the last case, the result is not conclusive since the glycan analyzed was obtained after hydrofluoric acid treatment, which could cleave the labile Gal*f.*

Antibodies directed towards Gal*f* epitopes have been obtained from GIPLs in rabbits [31]. A monoclonal antibody named 10D8 recognizes β-Gal*f* in the mucins of insect-stage strains from DTU I and some hybrid strains [82,83]; mucins from DTU II are not reactive. Moreover, a single-chain variable fragment (scFv) derived from mAb-10D8 was engineered to target the mucins of the metacyclic infective forms. It was proven that scFv-10D8 specifically inhibited parasite invasion of mammalian cells [84].

## 5. Structure of *O*-Glycans in Mucins of Epimastigotes and Metacyclic Trypomastigotes

In the mucins, the *O*-linked oligosaccharides may be derived from two cores, β-d-Gal*p* (**1**→**4**) GlcNAc (core 1) or β-d-Gal*f* (**1**→**4**) GlcNAc (core 2). Higher oligosaccharides, formed by elongation and/or branching with more galactoses, contribute to the microheterogeneity of the mucins. Only Gal*p* was found in oligosaccharides derived from core 1. Interestingly, oligosaccharides with core 2 may contain β-Gal*f* and β-Gal*p*.

On studying the fine structure of the glycans in several strains, it was concluded that core 2 was found in the mucins of strains belonging to DTU I isolated from sylvatic hosts, namely G strain [47,48], Colombiana [50], Dm28c [49] and Silvio X10/1 [85], and also in the hybrid Tulahuen strain [51], classified as DTU VI. A sialylated oligosaccharide was also identified in the mucins from Dm28c. The structures of the oligosaccharides in the mucins are shown in Table 1.

In the mucins of the Y strain belonging to DTU II (with a domestic transmission), only β-Gal*p* was found in the oligosaccharides, which was derived from core 1 (Table 2) [81]. On analyzing the oligosaccharides obtained from mucins of the Tulahuen strain and CL clones, included in DTU VI [3,94], controversial results were obtained. In the Tulahuen strain, both cores were identified, giving rise to a high diversity of oligosaccharides (Table 1). Besides the core 1 disaccharide, the trisaccharide resulting from the incorporation of sialic acid was identified, whereas the more complex chains in the Tulahuen mucins were derived from core 2 [51]. In the CL clones, only core 1 was identified, and the derived oligosaccharides lack Gal*f* and are similar to those found in the Y strain [19,46,52] (Table 2).

The role of the glycan structure of the GIPLs and mucins of epimastigotes in the interaction with the insect host was studied. Purified GIPLs from the Y strain inhibit the adhesion of epimastigotes to the insect midgut [95]. It was proven that, at least in part, the interaction with the midgut was due to the presence of Gal*f*.

The mucins oligosaccharides **1**–**9** of Table 1 were chemically synthesized as benzyl glycosides [22,86,87,88,89,90,91,92,93] and used for studies on the adhesion of the parasites to insect tissues [96]. Diverse results were obtained depending on the strain. It was shown that the branched (Gal*f*)-containing trisaccharide β-d-Gal*f*(**1**→**4**)[β-d-Gal*p*(**1**→**6**)]-α-d-GlcNAc is a determinant for the adhesion of Dm28c (DTU I) parasites to the rectal ampoule of the triatomine vector. Higher oligosaccharides bearing the same motif were even better competitive inhibitors. In contrast, the synthetic oligosaccharides did not show any inhibitory effect on the binding of CL Brener (DTU VI) epimastigotes to the triatomine hindgut, which is in agreement with the lack of Gal*f* in mucins of the CL strain [46]. Moreover, studies showed that the epimastigote mucins do not bind to the insect midgut [96], which however could attach GIPLs [95]. Although Gal*f* contributes to both interactions, these results point to different receptors for GIPLs and mucins. Adhesion to the rectal ampoule is the step before the differentiation into infective metacyclic trypomastigotes, which then detach from the cuticle. The process would be caused by changes in the composition of the surface molecules on metacyclogenesis [5,97]. However, in a comparative analysis of the GPI anchors and the *O*-linked chains from the mucins of epimastigotes and metacyclic trypomastigotes of the G strain, it was found that the only difference was in the lipid structure, which changes from an alkylacyl glycerol to a ceramide in the infective forms [47]. These results suggest that different moieties mediate the adhesion of epimastigotes and the release of the metacyclic forms.

## 6. Structure of Glycans in Mucins from Mammalian Cell-Derived Trypomastigotes. Immunogenicity of the α-Gal Epitope

**Figure 6:**
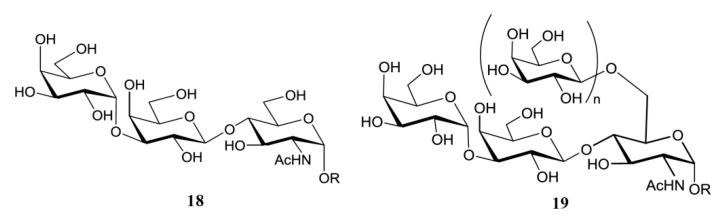
*O*-linked sugars in mucins of *T. cruzi* cell-derived trypomastigotes. **19** were assigned based on methylation analysis

The unique feature of the *O*-linked sugars in the mammal-stage parasites is the presence of the α-d-Gal*p* (**1**→**3**) Gal epitope. The hexosamine that links the carbohydrate to the polypeptide chain is the same in parasites of all stages and strains analyzed. Thus, the smallest oligosaccharide found in the mucins of mammal trypomastigotes was the trisaccharide α-d-Gal*p*(**1**→**3**)-β-d-Gal*p*(**1**→**4**)-GlcNAc (**18**) (Figure 6). Higher oligosaccharides were released from the mucins via a reductive β-elimination reaction [98]. Although the structure of the branched oligosaccharides was not fully described, the methylation studies pointed to branching at C-6 of the GlcNAc with Gal*p*, which should be in the β-configuration so as to function as an acceptor in the transfer reaction of sialic acid from host glycoconjugates [33,34,38]. This process is crucial for the pathogenesis of *T. cruzi*, since the lysis of trypomastigotes is prevented by sialylation [30,99]. The β-galactose in the branch may be further substituted with more β-Gal*p* (**19**) units, since 2,6-di-*O*-substituted Gal*p* was also identified in the methylation analysis. The highly relative amount of tetramethyl galactose detected in the mixture agrees with the presence of terminal Gal*p* necessary for the trans-sialidase reaction [98].

Variations in the expression of α-Gal in strains belonging to different DTUs were reported, with higher expressions of this sugar in Y *T. cruzi* populations, followed by Colombiana and CL strains [100].

The oligosaccharides are highly immunogenic due to the presence of the terminal α-Gal*p* units, which elicit the lytic anti α-Gal antibodies found in sera from patients with chronic Chagas disease. The α-d-Gal*p*(**1**→**3**)Gal epitope is common in the glycoconjugates of non-primate mammals, prosimians and New World monkeys, but is not found in Old World monkeys, apes or humans due to an evolutionary mutation, which resulted in the inactivation of the α-1,3-galactosyltransferase gene [101]. For that reason, healthy humans also produce α-Gal antibodies as a response to carbohydrate antigens from bacteria in normal gastrointestinal flora [102], however the response to the α-Gal epitope is much stronger in patients infected with *T. cruzi* [98,103].

Neoglycoconjugates containing the α-Gal epitope have been chemically synthesized and evaluated as diagnostic tools for Chagas disease, and also as candidates for therapeutic intervention [103,104,105,106,107,108,109]. It was shown that the trisaccharide α-d-Gal*p*(**1**→**3**)-β-d-Gal*p*(**1**→**4**)-β-d-Glc*p*, with glucose in the reducing end instead of GlcNAc, was as effective for the recognition of chagasic antibodies as the natural trisaccharide **18,** and easier to synthesize [103]. α-Gal immunity has been studied in other parasites, such as *Leishmania major* and *Plasmodium falciparum*. Neoglycoproteins containing α-Gal have been described as diagnostic tools for cutaneous leishmaniasis [110] and for protection against the parasite [111,112]. In *Plasmodium falciparum*, the agent of malaria, terminal α-linked galactosyl units, recognized by immune sera, are present in carbohydrate chains of glycoproteins [113,114].

## 7. Potential Role of Galectins in the Infection of *T. cruzi*

In view of the relation of the terminal β-galactopyranosyl units in the parasite to sialylation by TcTS, several groups have studied the role of galectins (Gal) in the infection, mainly focusing on Galectin **3**. The interactions of galectins with glycans were considered a fundamental event in pathogen recognition [115]. A mucin of 45kDa was quickly identified as a receptor for Gal **3** [116,117], and it was shown that a high Gal **3** expression enhances cell recognition of parasites [118,119]. Recent work showed that Gal **3** is important in the survival of the parasite during infection [120]. The role of Gal **1** in modulating the outcome of the infection was also studied [121,122]. In relation to the different glycan structures identified in the mucins depending on the strains and stages of *T. cruzi*, the work of Pineda et al. [25] investigated the binding of galectins **1**, **3**, **4**, **7** and **8** with 14 strains of DTUs I–VI of *T. cruzi* in different stages of its life cycle. The binding profile for the six DTUs agreed with the genetic classification. They found that the galectins bind preferentially to amastigotes. Although amastigotes lack mucins, β-galactopyranosides have been identified as determinants for host–cell interactions in the specific glycoprotein Ssp4 [59]. The fine glycan structure of Ssp4 was not reported. The higher recognition of amastigotes could be due to the lack of sialylation of the glycans, since no TcTS was detected in these forms. The authors did not find differences between cell-derived trypomastigotes and insect metacyclic trypomastigotes.

## 8. Conclusions and Perspectives

Carbohydrates in the surface of microbial pathogens have received, in the last few decades, special attention as targets for diagnosis and vaccine development. In T. cruzi, the problems encountered in generating mutants defective in glycosylation leads to chemical or chemoenzymatic methods for synthesizing the oligosaccharides and neoglycoconjugates for immunological studies. Several surface molecules in the parasite and in the host have been implicated in the adhesion and infection.

The two partners of a crucial interplay in *T. cruzi*, the mucins and the trans-sialidase, have been the most studied. They were called mucins because of the high content of sugar in their *O*-linked chains, although their structures differ significantly from the human mucins, as discussed in the text. One of the differences is that GlcNAc instead of GalNAc links the *O*-chain to the protein [41,42,44]. The lack of UDP-GlcNAc:polypeptide α-N-acetylglucosaminyl transferase in the intracellular amastigote stage points to the absence of mucins in these forms. Accordingly, no trans-sialidase (TS) genes were reported in these intracellular parasites [123]. One intriguing feature is the presence of galactose in three possible configurations, depending on strains and life stages. Gal*f* is only present in the stages that live in the insect vector, whereas α-Gal*p* is characteristic of blood trypomastigotes; both may generate specific antibodies for the structures that carry them. On the other hand, Gal*f* is not present in the mucins of all strains, and it was only found in DTU I and in the hybrid Tulahen strain (DTU VI) and is conserved when epimastigotes differentiate into metacyclic trypomastigotes. Some of the oligosaccharides of all strains and stages are branched with terminal β-Gal*p*, which provides the site for sialylation by the TcTS. The expression of TcTS is lower in metacyclic trypomastigotes than in mammal trypomastigotes; however, both the insect and the cell-derived trypomastigotes are able to invade mammalian cells. Sialylation differently affects parasites in each infective stage [83]. Sialyl external units in metacyclic forms impair the interaction with mammal cells. In fact, it was shown that the treatment of metacyclic *T. cruzi* of the G strain with neuraminidase increases the infectivity [124], whereas several groups have reported on the positive effects of the sialylation of blood trypomastigotes on the adhesion and invasion of mammal cells [29]. These reports point to different receptors in the mammal cells for both kinds of trypomastigotes. On the metacyclogenesis of epimastigotes, the mucin glycans conserve their structure, which have important differences from the mammal trypomastigotes. This topic deserves further investigations. β-Galactofuranosides are the sugar epitopes of some strains, and as far as we know no human lectins for Gal*f* have been described. The level of further substitution with β-Gal*p* units of the *O*-chains, determining a glycophenotype, may modulate the infection.

The Gal*f* in the branched oligosaccharides of epimastigote mucins is involved in the adhesion to the hindgut of the insect, where differentiation to the infective metacyclic forms takes place [96]. We also considered how to explain the adhesion of strains that lack Gal*f* in the mucins, like the Brenner strain. One possibility is that attachment is mediated by the Gal*f* in the GIPLs, since it is present in all the strains [77]. It was proven that upon starvation of UDP Gal*p*, Gal*f* incorporates into the GIPLs in preference over the mucins [54].

Several molecules on the parasite and the host are involved in mammal infection by metacyclic trypomastigotes. In fact, once in the mammal host, strains belonging to DTU I cause lower parasitemia titers than metacyclic forms from strains Y and CL, which do not contain Gal*f* in their mucins. The first question was if the presence of Gal*f* influences the sialylation of the β-Gal*p* present in the oligosaccharides. In vitro studies showed that Gal*f* does not interfere with sialylation [125]. If these results are extended to the in vivo process, it is possible that once in the cell, the Gal*f* may trigger the production of antibodies that impair infection. Moreover, the mucins of mammal trypomastigotes of representative stages lack Gal*f*, and it was reported that on the cell, metacyclic mucins are capped and extensively released in the parasitophorus vacuole [126]. The shedding of the mucins and other membrane components from different stages was reported [127], and this process may precede the construction of new mucins in blood trypomastigotes for adaptation to the new environments. In this respect, mucins were not detected in the intermediate amastigote stage.

The only monosaccharide transferase for mucins that has been identified is the UDP-GlcNAc:polypeptide α-N-acetylglucosaminyl transferase. An exo-β-galactofuranosidase has been purified from epimastigote lysates by affinity chromatography [78], but it was not fully characterized. This hydrolase could be important for the regulation of the interaction of the parasite with the insect tissues. The galactose transferases that introduce the galactoses in different configurations and linkages in the mucins, as well as the membrane receptors of the hosts, are interesting topics for further studies. The unique structures of the glycans point to them as selective targets for chemotherapy.

## Figures and Tables

**Table 1:** *O*-linked carbohydrates derived from core 2 in the mucins of *T. cruzi.*

Structure	Strain (Ref)	Chemical Synthesis (Ref)
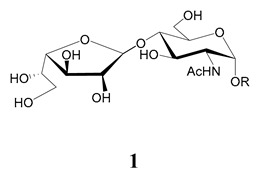	G [47,48] Dm28c [49] Colombiana [50] Tulahuen [51]	[86]
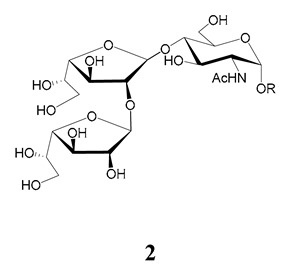	Tulahuen [51]	[87]
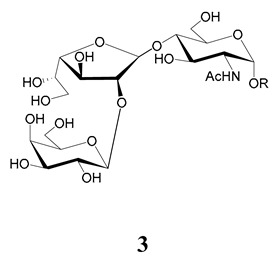	Tulahuen [51]	[87]
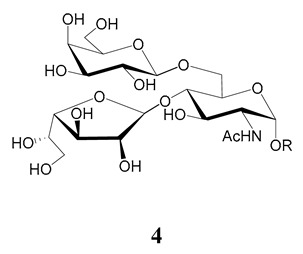	G [47,48] Dn28c [49] Colombiana [50] Tulahuen [51]	[88]
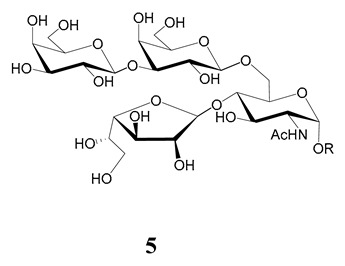	G [47,48] Dn28c [49] Colombiana [50] Tulahuen [51]	[89]
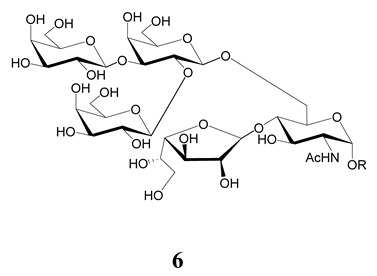	G [47,48] Dn28c [49] Colombiana [50] Tulahuen [51]	[90]
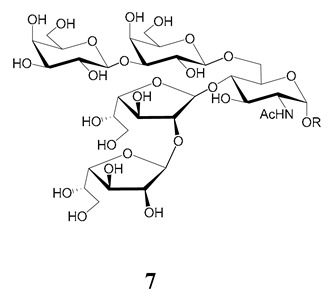	Tulahuen [51]	-
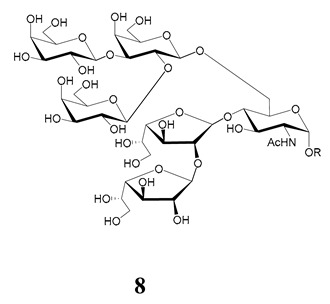	Dm28c [49] Tulahuen [51]	[91]
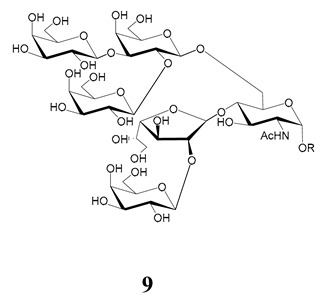	G [47,48] Dm28c [49] Colombiana [50] Tulahuen [51]	[92,93]
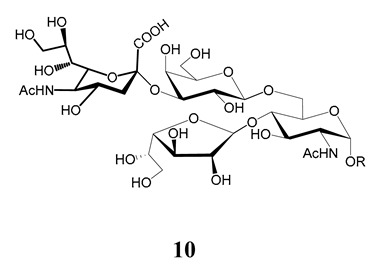	Dm28c [49]	-

**Table 2:** *O*-linked carbohydrates derived from core 1 in the mucins of *T. cruzi.*

Structure	Strain (Ref)
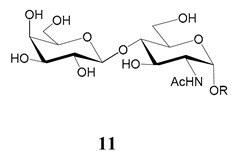	Y [81] CL Brener [19,46] Tulahuen [51] CL 14 [52]
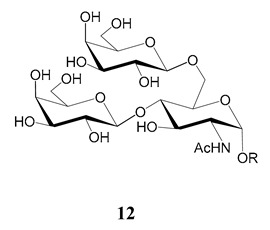	Y [81] CL Brener [19,46]
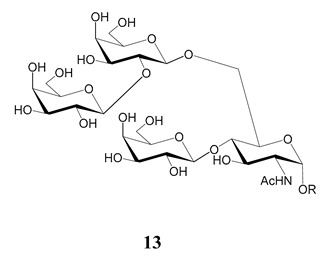	Y [81] CL Brener [46]
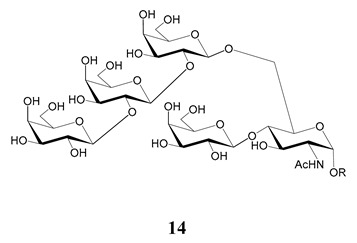	CL Brener [46]
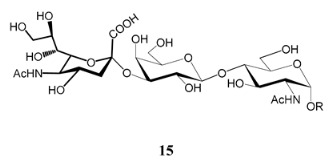	CL Brener [46]
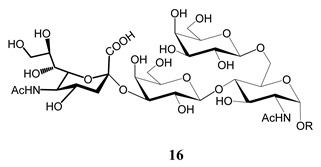	CL Brener [46]
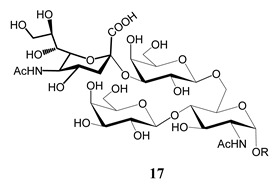	CL Brener [46]
